# Biohybrid Polymer-Antimicrobial Peptide Medium against *Enterococcus faecalis*

**DOI:** 10.1371/journal.pone.0109413

**Published:** 2014-10-03

**Authors:** Lea H. Eckhard, Asaf Sol, Ester Abtew, Yechiel Shai, Abraham J. Domb, Gilad Bachrach, Nurit Beyth

**Affiliations:** 1 Department of Prosthodontics, the Hebrew University – Faculty of Dental Medicine, Jerusalem, Israel; 2 Institute of Dental Science, the Hebrew University – Faculty of Dental Medicine, Jerusalem, Israel; 3 Institute for Drug Research, School of Pharmacology, Faculty of Medicine, the Hebrew University, Jerusalem, Israel; 4 Department of Biological Chemistry, the Weizmann Institute of Science, Rehovot, Israel; University of New South Wales, Australia

## Abstract

Antimicrobial peptides (AMPs) are conserved evolutionary components of the innate immune system that are being tested as alternatives to antibiotics. Slow release of AMPs using biodegradable polymers can be advantageous in maintaining high peptide levels for topical treatment, especially in the oral environment in which dosage retention is challenged by drug dilution with saliva flow and by drug inactivation by salivary enzymatic activity. *Enterococcus faecalis* is a multidrug resistant nosocomial pathogen and a persistent pathogen in root canal infections. In this study, four ultra-short lipopeptides (C16-KGGK, C16-KLLK, C16-KAAK and C16-KKK) and an amphipathic α-helical antimicrobial peptide (Amp-1D) were tested against *E. faecalis*. The antibacterial effect was determined against planktonic bacteria and bacteria grown in biofilm. Of the five tested AMPs, C16-KGGK was the most effective. Next C16-KGGK was formulated with one of two polymers poly (lactic acid co castor oil) (DLLA) or ricinoleic acid-based poly (ester-anhydride) P(SA-RA). Peptide-synthetic polymer conjugates, also referred to as biohybrid mediums were tested for antibacterial activity against *E. faecalis* grown in suspension and in biofilms. The new formulations exhibited strong and improved anti- *E. faecalis* activity.

## Introduction

The widespread use of antibiotics leads to the emergence of more resistant and virulent strains of microorganisms. Consequently, the development of new antimicrobial agents becomes paramount for novel treatment options [1]. Moreover, bacterial resistance to antibiotics becomes even more complicated when dealing with bacterial biofilms. Interestingly, potent antimicrobial components against a wide range of pathogens can be found in the innate immune system of various organisms including humans. An example is host-defense cationic antimicrobial peptides (AMPs) which are conserved evolutionary components that possess the capacity to kill invading microbes [2]. It is generally accepted that AMP-mediated killing typically occurs through microbial membrane disruption resulting in irreparable damage.

AMPs exhibit broad-spectrum activity against a wide range of microorganisms including Gram-positive and Gram-negative bacteria, protozoa, yeast, fungi and viruses [4]. Furthermore, whereas conventional antibiotics are becoming less effective, bacteria do not appear to develop resistance to AMPs.

AMPs function and mode of action is a direct derivative of their structure and electric charge. They differ in amino acid length (12–50 aa), sequence and dimensional structure, they are composed of about 50% hydrophobic amino acids and their electric charge is positive. This unique amphipathic structure allows AMPs to bind to the negatively charged outer surfaces of microorganisms and to disrupt and permeate their cell membranes [3]. Consequently, their advantage over conventional antibiotics is a non-specific but antimicrobial selective mode of action. Several resistance strategies to avoid AMPs function were reported. These included, degradation with extracellular proteases [4], altering the net surface charge [5], transporting AMPs into the cytoplasm and degrading them [6] and exporting AMPs by efflux pumps [7].

AMPs are one of the reasons why humans stay healthy [2]. In humans, one of the known potent AMPs groups is the defensins. Specifically, human beta-defensin 3 (hBD3). hBD3 is considered the most potent β-defensin peptide described so far [8]. An additional group of potent antimicrobial peptides, produced in bacteria and fungi are the lipopeptides. They are composed of specific lipophilic moieties attached to anionic peptides. Unfortunately, native lipopeptides are non-cell selective and thus can be toxic to mammalian cells. Interestingly, all of the structural advantages of the native AMPs can be recruited to synthesize improved antimicrobial agents, *e.g.* ultra-short lipopeptides and amphipathic α-helical antimicrobial peptide (Amp-1D). Recently, it was reported that ultra-short lipopeptides composed of only four amino acids conjugated to an aliphatic acids chain (16C, palmitate) can achieve potent antimicrobial activity without compromising biocompatibility.

*Enterococcus faecalis* is a microorganism residing in the gastro-intestinal tract. None the less, *E. faecalis* can cause life-threatening infections such as: endocarditis, bacteremia, urinary tract infection and meningitis. These complications are mostly associated with the acquisition of resistance to antibiotics. In dentistry, *E. faecalis* is considered a persistent root canal pathogen [9].

Integration of the structural and functional properties of peptides and proteins with the versatility of synthetic polymers has gained significant interest in material design and application [10]. Peptides and proteins have unique structures that convey their ability to function in specific biological activities. Hybrid molecules of peptides conjugated to polymers can be used for various applications with the advantages of being resistant to enzymatic cleavage and less cytotoxic to human cells [11]. Peptide-synthetic polymer conjugates, also referred to as biohybrid medium, consist of biologically relevant peptides and synthetic polymers, aiming to combine the advantages of the two components, namely biological function (biological component) and process-ability (synthetic component). A slow release mechanism can enable high concentration maintenance of therapeutic agents for prolonged periods of time. Examples of biodegradable polymers that were previously described as efficient controlled delivery mediums include the fatty acid-based polymer poly (lactic acid co castor oil) and the ricinoleic acid-based poly (ester-anhydride) [11]–[15]. Herein, biohybrid medium consisted of two different assembled components, including a polymer matrix, which was responsible for the sustained release function and an antimicrobial agent, *i.e.* AMPs, which was responsible for the potency of the formulation. In the present study novel formulations of biodegradable polymers integrating AMPs were evaluated against *E. faecalis* in planktonic bacteria and biofilm growth.

## Materials and Methods

### Test materials

#### Antimicrobial peptides

Human recombinant β-defensin 3 (hBD3) (GIINTLQKYY CRVRGGRCAV LSCLPKEEQI GKCSTRGRKC CRRKK) was obtained from PeproTech (Lot #0108210, Rocky Hill, NJ, USA). Five different synthetic AMP candidates were tested. amphipathic α-helical antimicrobial peptide (Amp-1D) and four ultra short lipopeptides which were synthesized, purified and confirmed as described before [16], [17].

#### Biodegradable polymer synthesis

Poly (lactic acid co castor oil) (DLLA) and ricinoleic acid-based poly (ester-anhydride) P(SA-RA) were synthesized as previously describe [11]–[13], [15], [18]. Briefly, a poly (ester-anhydride) copolymer of sebacic acid (SA) and ricinoleic acid (RA) in a 3∶7 w/w ratio [P(SA-RA) 3∶7] was synthesized by transesterification followed by anhydride melt condensation. SA was used as supplied by Sigma-Aldrich (St. Louis, MO, USA) without any additional purification. RA (>98%) was isolated from castor oil by fractional precipitation based on salt-solubility. Poly (DL lactic acid co castor oil) 4∶6 and 3∶7 designated P(DLLA:CO) 4∶6 and P(DLLA:CO) 3∶7 was prepared using racemic mixture (DL) lactic acid. The synthesized polymers were characterized by Infrared (IR) spectroscopy and nuclear magnetic resonance (NMR) spectroscopy. Gel permeation chromatography (GPC) was used to estimate the molecular weight.

#### Formulation of AMP-based biohybrid media

The peptide powder was mixed with a pasty polymer to form a uniform homogeneous paste. A novel formulation of peptide and biodegradable polymer was prepared at a ratio of 100 µg peptide integrated in 100 mg polymer. The two ingredients were mixed manually with a spatula.

### Preparation of bacterial suspension

*E. faecalis* (ATCC #v583), was cultured overnight in 5 ml brain-heart infusion (BHI) (Difco, Detroit, MI, USA) broth supplemented with 2 mg/ml vancomycin (Sigma-Aldrich), at 37°C under aerobic conditions. The top 4 ml were transferred to a fresh test tube and the optical density (OD) was determined according to the specific experiment.

### Antibacterial activity

#### Minimal inhibitory concentration

The antibacterial activity of the peptides was examined using the microdilution assay [19]. Briefly, the bacterial suspension (at OD 0.3) was diluted at a ratio of 1∶1000. Aliquots of 150 µl of bacterial suspension were added to 50 µl of peptide dilutions in phosphate buffered saline (PBS) (Sigma-Aldrich) (in triplicate for each concentration) in a 96-well plate (Nunc 96 microtiter plates, Roskilde, Denmark). The optical density (595 nm) in each well was recorded every 20 min using a microplate reader (VERSAmax tunable microplate reader, molecular devices, Sunnyvale, CA, USA) at 37°C for 18–24 hrs. The minimal inhibitory concentration (MIC) was determined as the concentration which inhibited visible growth after 18–24 hrs.

#### Antibacterial activity of controlled released peptide

A total 10 mg of formulation was placed on the side walls of each of 6 wells in a 96 microtiter plate and then 270 µl of medium (BHI supplemented with vancomycin) were added Every 24 hrs the medium was collected and transferred to a new set of 6 wells in the same 96-well-plate and fresh medium was added to the 6 original wells containing the tested formulation. After one week, a 10 µl volume of *E. faecalis* suspension was added to each of the 6 wells and bacterial outgrowth was recorded. The plate was incubated at 37°C in a VERSAmax microplate reader and turbidity (OD_650 _nm) changes were recorded, every 20 min for 18–24 hrs.

### Antibiofilm activity

Antibiofilm activity was tested on *E. faecalis* biofilms grown for 72 hrs. Biofilm was formed in microtiter plates (24 well plates for the ATP bioluminescence assay and 96 well plates for the crystal violet biomass assay and confocal laser spectroscopy). Saliva was collected from one donor and DL-Dithiolthreitol (DTT) (Thermo Scientific, Abu-Gosh, Israel) was added to 2.5 mM. The suspension was kept at 4°C for 10 min and then centrifuged for 15 min at 6,500×g. The supernatant was transferred to a fresh sterile tube and diluted to 25% with sterile double distilled water (DDW). The diluted saliva was disinfected using a 0.2 µm vacuum-driven filter (0.22 µm, 250 µl, Jet biofil, Belgium). Wells in the microtiter plate were coated with clarified saliva by adding the saliva to the wells for 1 hr at 37°C (150 µl of saliva in the 24 well plate and 50 µl in the 96 well plate). Unbound saliva was removed and the wells were washed gently with PBS. The polymer peptide formulations were placed on the side walls of the wells and 10 µl of bacterial suspension (prepared as described above) were placed in the center of each well not touching the coated sidewall. The saliva coating was used to cover the entire well surface, followed by formulation placement on the sidewall of the wells. The bacterial inoculum was placed in the center of each well, not touching the formulation. After 1 hr incubation at 37°C BHI broth was added (1 ml in the 24 well plate and 100 µl in the 96 well plate). BHI broth was added every 24 hrs during 72 hrs. After 3 days the medium was discarded and the wells were washed gently with PBS. Bacterial metabolism in the attached biofilm was assessed using ATP bioluminescence. Biofilm mass was measured using crystal violet as described below.

#### ATP bioluminescence

Bacterial killing was evaluated by measuring intracellular ATP levels, an energy parameter commonly used as an indicator of cell injury and viability [19]. The 72 hr biofilm formed on the bottom of the wells was scraped using a pipette tip and collected into a set of 15 ml tubes. The cells were then centrifuged (6,500×g, 5 min), resuspended in 1 ml Lysis Buffer (2 mM DTT, 2 mM trans 1,2 Diaminocyclohexane NNNN Tetraacetic acid, 0.5 mM EDTA, 1% Triton, 25 mM Tris, 25 mM K_2_HPO_4_, 10% glycerol) and transferred to a 2 ml microcentrifuge tube containing glass beads (Lysing Matrix tubes, 0.1 mm silica spheres; MP Biomedicals, Eschwege, Germany). The cells were disrupted with the aid of a FastPrep cell disrupter (MP Biomedicals, Irvine, CA, USA). The tube was centrifuged for 10 min (4°C, 13,400×g). ATP levels were determined using an ATP bioluminescence assay kit (CLS 2, Roch Diagnostics, Mannheim, Germany). In a 96 microtiter plate designed for luminescence assay (Thermo Scientific, NUNC, 96-well optical Btm Plt white, Rochester, NY, USA) a 100 µl volume of the samples was added to 6 wells for each tested group. Then 100 µl luciferase (from the kit) were added to the same wells. The plate was inserted in a GENios reader (TECAN, Salzburg, Austria) and luminescence was measured using the Magelan program (TECAN, V6.6, 2009). ATP calibration was performed using ATP and luciferase from the kit.

#### Crystal violet

The total biofilm yield was assessed using crystal violet staining as follows. Biofilm fixation was performed using 200 µl methanol (MERCK, Darmstadt, Germany) that were added to each well for 20 min. The biofilm was then stained using 200 µl 1% crystal violet (Merck) for 20 min. Then the wells were washed gently 3x with PBS, and 200 µl of 30% acetic acid (GADOT, Netanya, Israel) were added to the wells. The acetic acid was transferred to wells of a new 96-well microtiter plate that was placed in a microplate reader and absorbance (OD_595 _nm) was measured.

#### Confocal microscopy

Confocal laser scanning microscopy (CLSM) was used to explore the vitality of bacteria in the different depth layers of the biofilm. Bacteria were stained using a live/dead kit (Live/Dead BacLight viability kit, Molecular Probes, OR, USA) as described before [20]. Briefly, wells were washed, incubated for 15 min in a solution containing propidium iodide and SYTO 9 and washed again. To read the results directly, the wells were coated with emulsion oil to prevent dehydration. Fluorescence emission was detected using a Zeiss LSM 410 confocal laser scanning microscope (Carl Zeiss Microscopy, Jena, Germany). Red fluorescence was measured at 630 nm and green fluorescence at 520 nm; objective lenses: x60/oil, 1.4 numerical aperture. Horizontal plane (x-y axes) optical sections were made at 700 µm intervals from the surface outwards and images were displayed individually. The biofilm was quantified by measuring the area occupied by the bacteria with the aid of Image Pro 4.5 software (Media Cybernetics, Rockville, MD, USA).

### Statistical analysis

The presented data are the mean and standard deviation of triplicates of a representative experiment repeated three times. The growth mean, and multiple comparisons of growth inhibition by AMP (compared with the growth of untreated bacteria) were calculated from each growth curve using Student’s t-test. The level of significance was p<0.01.

## Results

### Antibacterial activity

#### Minimal inhibitory concentration

**Figure 1:**
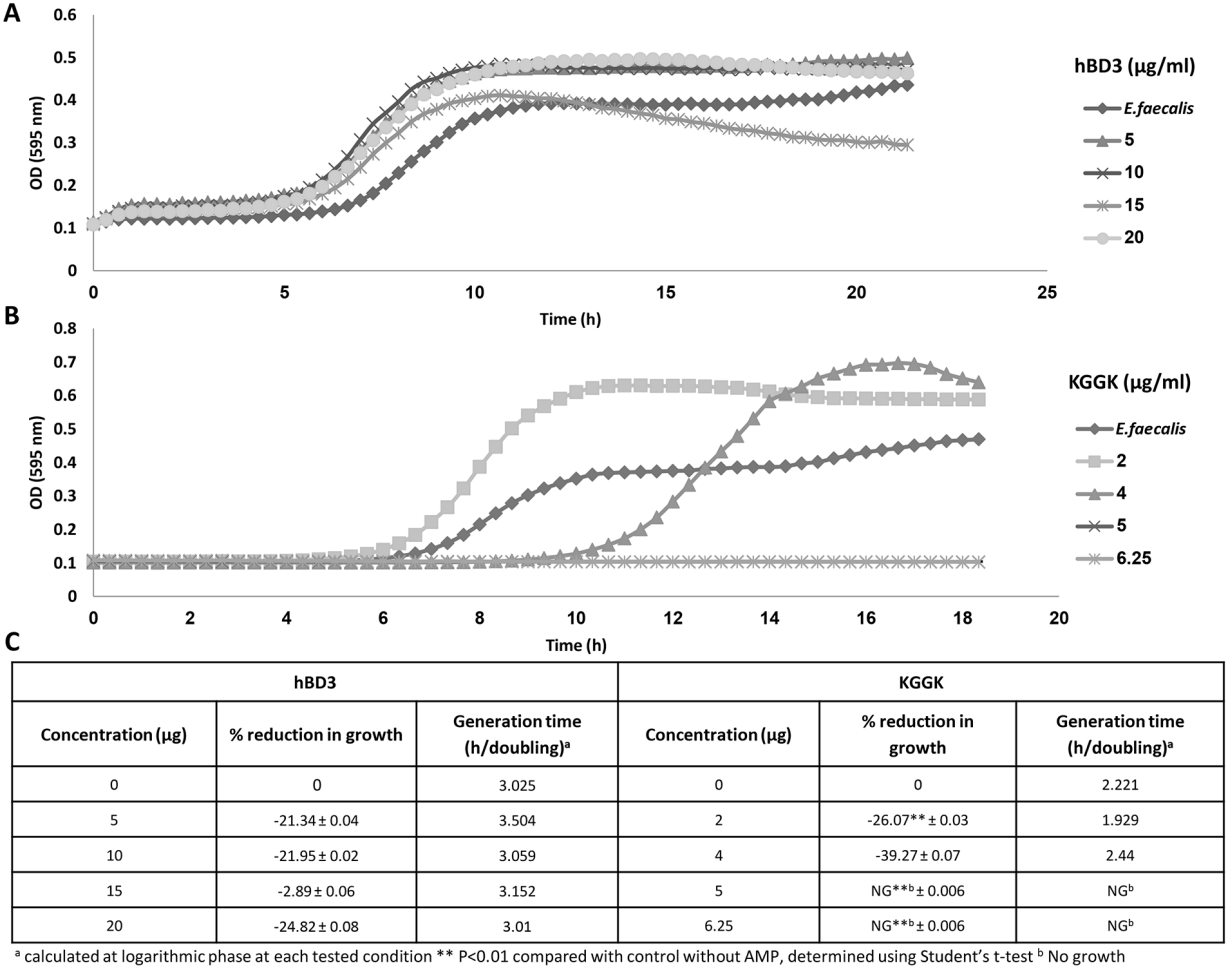
*E. faecalis* growth is inhibited by C16-KGGK but not by hBD3. Growth of *E. faecalis* was measured (see Materials and Methods) in the presence of increasing concentrations of hBD3 (A) or of the lipopeptide KGGK (B). Percent growth inhibition was calculated compared with that of untreated bacteria during the logarithmic phase of the non treated bacteria. Generation time was calculated from each curve using the section representing the exponential growth phase (C).

The MICs results for each of the tested AMPs are summarized in Table 1. Growth of *E. faecalis* was not inhibited by hBD3 at concentrations of up to 20 µg/ml. Amp-1D did not affect bacterial growth at concentrations of up to 25 µg/ml. C16-KGGK, C16-KKK, C16-KAAK and C16-KLLK completely inhibited bacterial growth at concentrations ranging between 5 and 25 µg/ml. The most potent AMP was the lipopeptide C16-KGGK that caused complete growth inhibition at 5 µg/ml (Fig. 1). As a result, all further formulations were tested using the C16-KGGK lipopeptide. Surprisingly, at concentrations below 5 µg/ml the growth of *E. faecalis* was not inhibited by C16-KGGK but was actually accelerated.

**Table 1:** Anti *E. faecalis* MICs of the AMPs investigated.

AMP	Amino acid sequence	MIC [µg/ml]
hBD3	GIINTLQKYY CRVRGGRCAV LSCLPKEEQI GKCSTRGRKC CRRKK	>20
Amp-1D	LKLLKKLLKKLLKLL-NH_2_	>25
C16-KGGK	CH_3_(CH_2_)_14_CO–KGGK-NH_2_	4–5
C16-KKK	CH_3_(CH_2_)_14_CO–KKK-NH_2_	6–12.5
C16-KAAK	CH_3_(CH_2_)_14_CO–KAAK-NH_2_	12.5–25
C16-KLLK	CH_3_(CH_2_)_14_CO–KLLK-NH_2_	6–12.5

Underlined amino acids are D-enantiomers.

#### Sustained release and anti-*E. faecalis* activity

**Figure 2:**
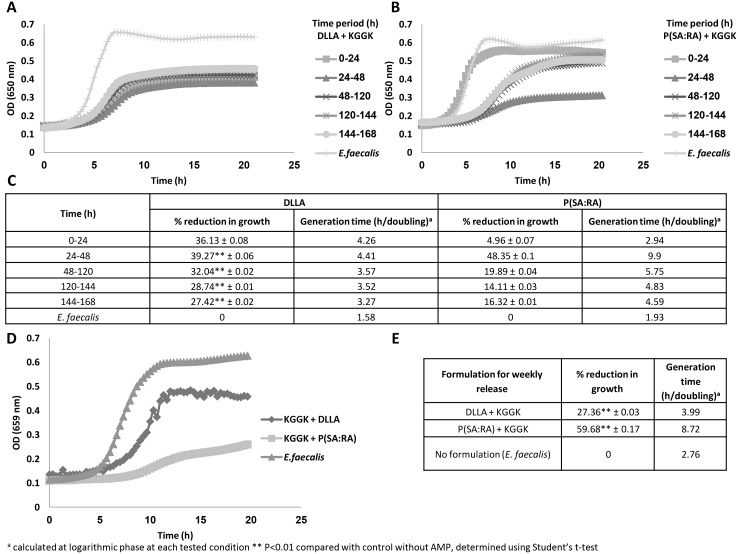
Growth inhibition of *E. faecalis* by KGGK released from P(SA:RA) or from DLLA. The side walls of 6 wells from line A of a 96 microwell plate were coated with the tested formulation (100 μg peptide+100 mg polymer, ratio 1∶1000). Fresh medium was added to the first line of wells and was transferred every 24 hrs to a new line below for a week. Then the bacteria were added to the tested wells and the plate was incubated at 37°C in a VERSAmax microplate reader and OD_650_ in each well was followed automatically for 20 hrs. (A, C) KGGK+DLLA. (B, C) KGGK+ P(SA:RA) (D, E) weekly release of both formulations. Percent growth inhibition calculated compared with that of the non- treated bacteria during the logarithmic phase of the non treated bacteria. Generation time was calculated from each curve using the section representing the exponential growth phase (C, E).

Release of the C16-KGGK lipopeptide from two biodegradable polymers was monitored over one week in two modes. In the first, the antibacterial action of C16-KGGK released into the medium that came in contact with the formulation every 24 hrs was measured (see Fig. 2A, B). In the second, the bacteria were added to the wells with C16-KGGK that was released from the polymer and accumulated for one week (see Fig. 2C). The anti*-E. faecalis* activity of C16-KGGK released from each formulation was reflected by: 1. The final optical density of the treated bacteria, which was lower than that of the untreated ones. 2. The slope of the curve (generation time, see Fig. 2C), which was more moderate in the treated bacteria.

The anti*-E. faecalis* activity in the medium exposed to the formulation for an entire week (see Fig. 2D, E) generated a longer generation time, especially with P(SA:RA). Bacteria treated with the DLLA formulation exhibited a 27% reduction in growth and those treated with P(SA:RA) a 60% reduction compared with the non treated bacteria.

### Anti-biofilm effect

#### Crystal violet dye

**Figure 3:**
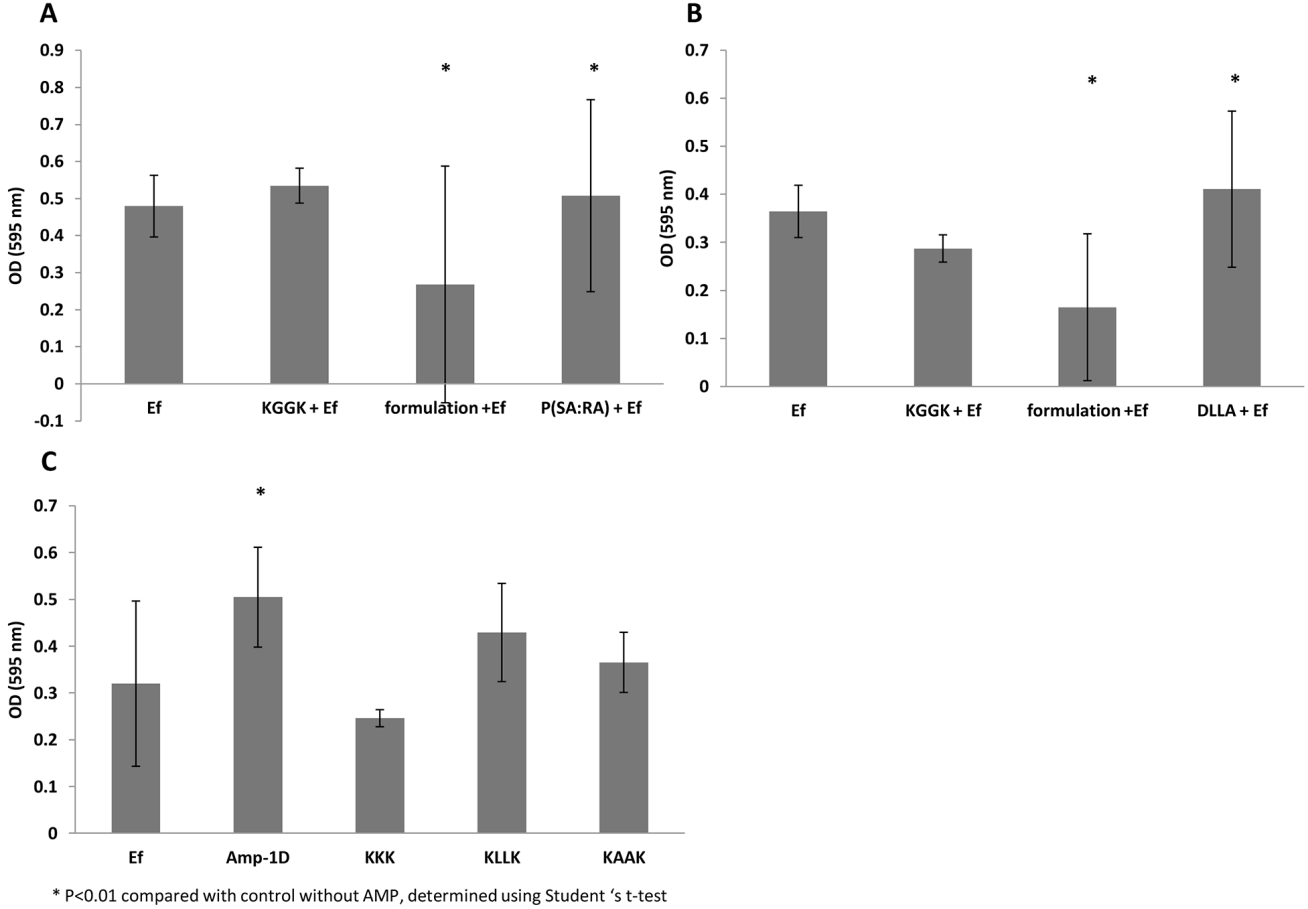
Effect of the antimicrobial peptides on the development of *E. faecalis* biofilms. *E. faecalis* biofilms were grown in 96 microtiter plate wells for 72 hrs in the presence of KGGK formulated with P(SA:RA) (panel A), or formulated with DLLA (B,) or with soluble peptides (C). *Ef* represents the non-treated bacteria, *KGGK+EF* - bacteria treated only with peptide, *formulation+EF -* bacteria treated with polymer and peptide and *Ef + polymer -* bacteria treated only with polymer control). The biofilm was stained with 1% crystal violet measured at OD 595 nm (see Materials and Methods). The optical density of the polymers alone without the bacteria was subtracted from the results of the biofilm that came in contact with the formulation and the polymer.

Crystal violet was used to stain and measure biofilm mass so that inhibition of biofilm formation in the presence of C16-KGGK formulated with P(SA-RA) (Fig. 3A) or DLLA (Fig. 3B) or in the presence of the soluble tested AMPs (Fig. 3C) could be determined. A significant anti-biofilm effect was obtained with C16-KGGK using both formulations but not with the soluble C16-KGGK (Fig. 3A–B). The vehicle formulation itself does not possess anti-biofilm activity. From the other peptides tested in suspension, only C16-KKK showed anti-biofilm activity (Fig. 3C).

#### ATP bioluminescence assay

**Figure 4:**
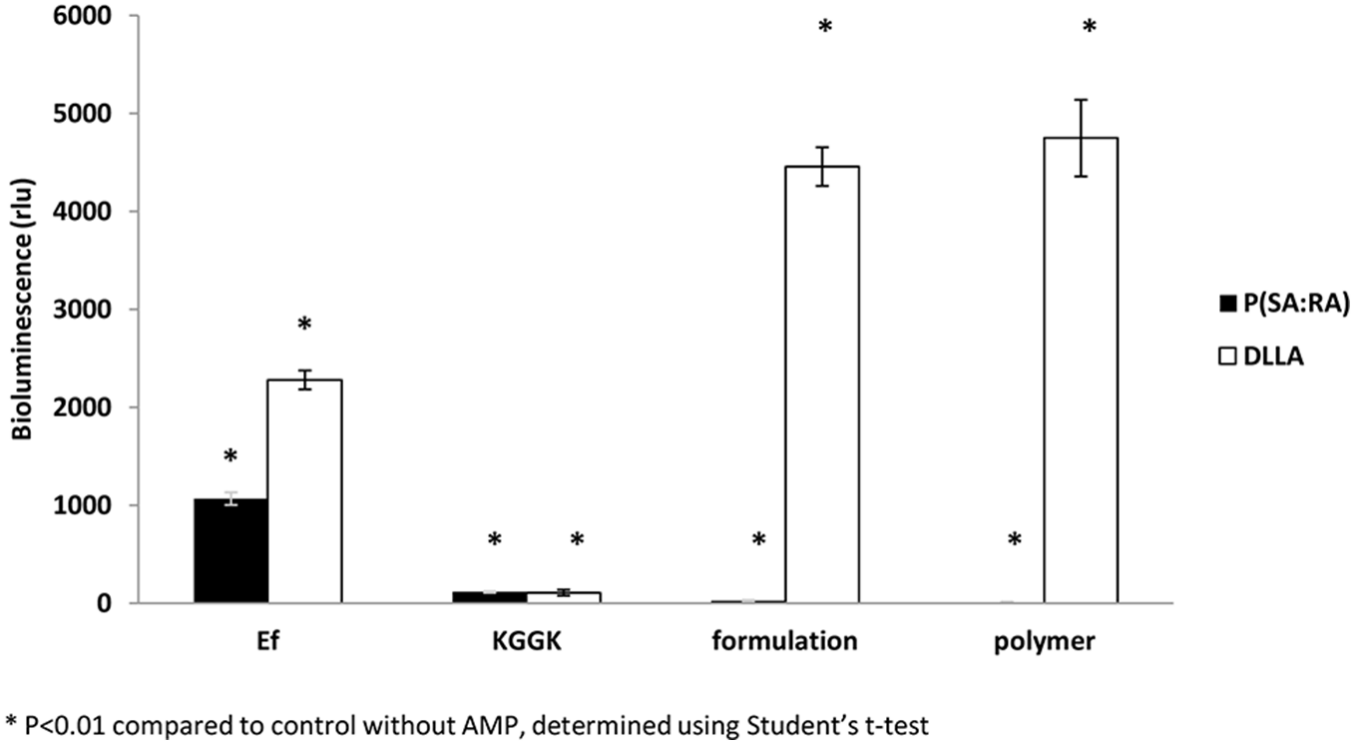
Effect of KGGK incorporated in biodegradable polymer on ATP in *E. faecalis* biofilm. Biofilm was exposed to the formulation for 72 hrs and ATP was measured as described in Materials and Methods. *Ef* represents the untreated bacteria as control, *KGGK* - bacteria treated only with peptide*; formulation -* bacteria treated with sustained release peptide; *polymer -* bacteria treated only with polymer as control.

The level of ATP indicates the active metabolism of a cell. ATP levels in *E. faecalis* biofilms treated with soluble C16-KGGK were relatively low compared with that in the untreated control (*E. faecalis* alone) (see Fig. 4). The P(SA:RA) formulation (without C16-KGGK) also reduced bacterial viability. As opposed to P(SA:RA), DLLA had the reverse effect and the luminescence values were much higher than that of the positive control. These findings led to the question whether the luminescence values are derived from bacterial number, the metabolic status or both. In addition, a similar experiment was performed in which the biofilm was first grown for 48 hrs and then the tested materials were added to verify if C16-KGGK can affect an already constructed biofilm. The results were similar to those above (where the materials were added immediately after inoculating the bacteria). This may indicate that the formulation and the peptide (each) have an anti-metabolic effect even after the biofilm is formed.

#### Bacterial vitality

**Figure 5:**
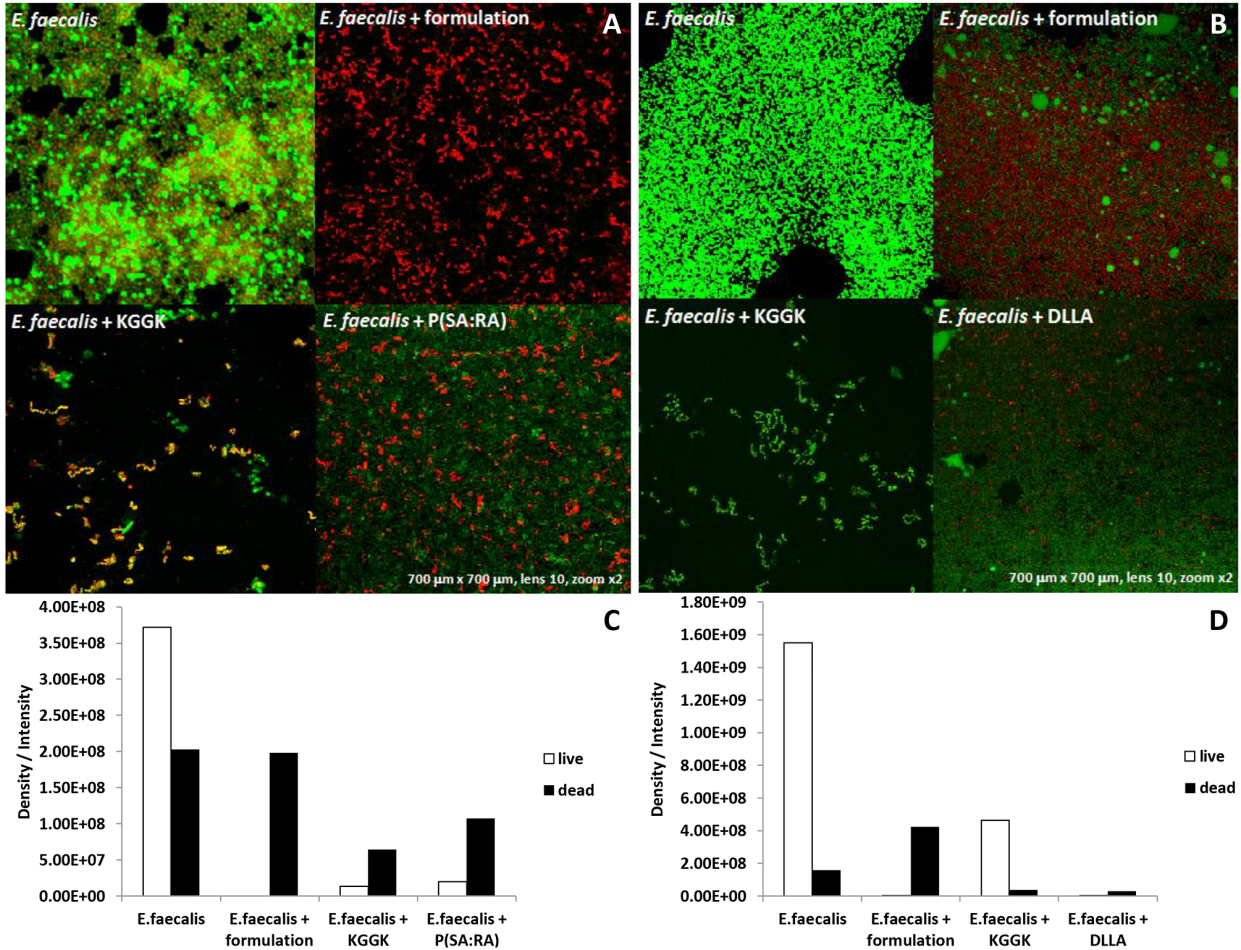
Live/dead assay. *E. faecalis* came in contact with the examined materials for 72 hrs to form biofilm. The medium was discarded and the wells were washed gently with PBS. The live bacteria were stained with green dye, the dead bacteria were stained with a red dye. A 5 ml volume of each dye from the dead/live dying kit was added to 450 µl PBS using an Eppendorf and 30 µl of the solution were added in each well. Images were taken using an Olympus confocal microscope [A, B]. The black column represents the dead bacteria, the white column represents the live bacteria. The biofilm was quantified by measuring the area occupied by the bacteria with the aid of Image Pro 4.5 software (Media Cybernetics) [C, D].

To test the vitality of the bacteria within the biofilm by a different, independent method, live/dead staining followed by confocal microscopic analysis was performed. The differences between the four tested groups are clearly evident for both P(SA:RA) and DLLA incorporated C16-KGGK (Fig. 5). Soluble C16-KGGK induced death in the biofilm bacteria (Fig. 5A–B, *E. faecalis* + KGGK). However, C16-KGGK in both formulations was more effective than the soluble peptide alone. The P(SA:RA) polymer had a strong inhibitory activity against biofilm formation as seen by the reduction in bacterial load.

## Discussion

Antimicrobial peptides are one of nature’s solutions to bacterial invasion. Their nonspecific mode of action, which is based on physical membrane disruption, is effective against various bacteria and is less likely to induce bacterial resistance than antibiotics. Recently, synthetic AMPs mimicking these strategic antibacterial agents have been gaining interest. Combining sustained release and an antimicrobial compound holds many advantages and has proved itself in the past. In this study a potent antimicrobial agent was identified against *E. faecalis*, and then incorporated in two candidate biodegradable polymers. The most efficient of the six investigated antimicrobial peptides was the lipopeptide C16-KGGK.

The antibacterial effect in this study was tested against *E. faecalis*. We chose this bacterium as an example of a pathogen that causes severe nosocomial infections and as an example of a strongly forming biofilm bacterium. *E. faecalis* can grow and survive in a wide range of environments (wide range of temperatures and pH) affording it the ability to surmount many obstacles [9]. Interestingly, root canal treated teeth are about nine times more likely to harbor *E. faecalis* than are primary infections. *E. faecalis* has been found in root canal-treated teeth in 30% to 90% of the cases. This frustrating rate of post treatment disease is mainly attributed to the limitations of the present technology that offers no tool to combat intra-canal infection following the cleaning and shaping stage of the endodontic treatment [21].

The tested antibacterial peptides were first assayed in suspension against planktonic *E. faecalis*. Although hBD3 was previously reported as being a highly potent antibacterial AMP against *E. faecalis* [8], [22]–[24], in the present study it showed an antibacterial effect against *E. faecalis* only when used at high concentrations. This may be due to the differences in *E. faecalis* strains and the hBD3 chemical synthesis. As hBD3 is a costly peptide, high concentrations are predestined to be irrelevant as a conventional therapeutic agent and thus were not tested further. Screening of the AMPs’ MICs demonstrated that the C16-KGGK lipopeptide was the most potent against *E. faecalis* and it was further investigated and formulated into biodegradable polymers. Interestingly, in some experiments at low concentrations bacterial growth was not inhibited but rather accelerated. As this phenomenon may compromise the antimicrobial effect, further investigation of the peptides’ mode of release is required. The exact mechanism of this opposite outcome is unknown, but the main assumption is that somehow the bacteria overcome lower concentrations of the lipopeptide and show accelerated growth compared with the untreated bacteria. This phenomenon needs to be considered when dealing with the amount of peptides that are released from the polymer. The new biohybrid medium incorporating C16-KGGK results in an anti- *E. faecalis* effect when tested against planktonic bacteria. Indeed calculation of bacterial number (using a calibration curve) revealed that the final bacterial load was lower by one order of magnitude in the treated wells. Additionally, the slope of the curve representing the bacterial growth rate (generation time) was more moderate in the treated bacteria, showing that the peptide is released into the medium. The generation time of the bacteria treated with each of the formulations and especially with P(SA:RA) was longer compared with that of the non-treated bacteria.

Bacteria grow naturally as biofilm, especially *E. faecalis* within the root canal. Moreover, *E. faecalis* is known to form biofilms that greatly increase its resistance to phagocytosis, antibodies and antimicrobials [21]. Therefore, in the second part of the study the anti-biofilm effect was tested. Three approaches were used to test the activity of the soluble AMPs and the new controlled release C16-KGGK lipopeptide formulations against *E. faecalis* biofilms. In the first, crystal violet was used to stain and measure biofilm mass. In the second, an ATP bioluminescence assay was performed and used as a viability indicator. In the third, the vitality of bacteria grown in a biofilm was tested using a dead/live stain. All three experiments revealed inhibition of biofilm formation when *E.* faecalis was exposed to the novel formulation. The three aspects examined were the amount of biofilm, its metabolic state and bacterial viability. Interestingly, the formulations were effective against a biofilm in the process of formation and against an established biofilm (mature biofilm). This is an important finding considering the fact that mature biofilm is much harder to treat because of its virulence factors. Moreover, we specifically tested the formulations’ potency against ATCC v583 strain due to its high known resistance to several antibiotics (among them vancomycin), compared to other strains such as ATCC 29212 [25]. It can be suggested that a formulation that was shown to be active against ATCC v583 is likely to be potent against other *E. faecalis* strains.

The polymer candidates which contain fatty acids have several advantages over other biodegradable polymers such as: flexibility, low melting point, improved handling and provide better degradation and release profiles [26]. As previously reported, biodegradable polyanhydrides and polyesters are useful materials for controlled drug delivery. They have a hydrophobic backbone with hydrolytically labile anhydride and/or ester that may be hydrolyzed to dicarboxylic acids and hydroxy acid monomers when placed in an aqueous medium. Fatty acids are suitable candidates for the preparation of biodegradable polymers, as they are natural body components and hydrophobic, and thus may retain an encapsulated drug for longer time periods when used as drug carriers [11]. Moreover, it was shown that these polymers are biocompatible [15]. As described before, this polymer-peptide interaction may hold many advantages over the peptide by itself, such as improved solubility, reduced immunogenicity, increased stability against degradation and prolonged biological activity [27]. Two different polymers were tested as delivery media and led to different results in their activity and mode of action. In the sustained release experiments, DLLA showed similar bacterial kinetic growth curves whereas P(SA:RA) did not, indicating that the two have separate modes of release. Furthermore, in the ATP bioluminescence assay, DLLA presented higher levels of luminescence and accordingly higher levels of ATP, suggesting that this polymer elevates the metabolic state of the biofilm, compared with P(SA:RA) which had the opposite effect. In the live/dead assay, the main difference between the two polymers is that in P(SA:RA) a larger amount of dead bacteria appeared, reinforcing our previous findings that P(SA:RA) itself may be an antibacterial agent. Thus, P(SA:RA) is apparently a more suitable delivery medium for this purpose.

Within the sustained release field wider experiments should be performed in order to learn the exact amount of peptide released from the polymer and the kinetics of the release. Furthermore, the period tested in this study was one week so that additional experiments should be performed using various periods of time.

## Conclusions

Synthetic AMPs were shown to have an effective antimicrobial activity against *E. faecalis*. A peptide that allows selective killing of *E. faecalis* would be a good candidate for endodontic treatment. Here, we show that a synthetic lipopetide can be highly effective against *E. faecalis*. Moreover, this lipopeptide when formulated in a biohybrid polymer medium has an increased antibiofilm effect. Thus, the novel effective formulation presented here can be advantageous in root canal treatment for the prevention of endodontic failure due to *E. faecalis*.

## References

[1] Blondelle SE, Perez-Paya E, Houghten RA (1996) Synthetic combinatorial libraries: novel discovery strategy for identification of antimicrobial agents. Antimicrob Agents Chemother 40: 1067–1071. 8723442 10.1128/aac.40.5.1067 PMC163267

[2] Boman HG (2003) Antibacterial peptides: basic facts and emerging concepts. J Intern Med 254: 197–215. 12930229 10.1046/j.1365-2796.2003.01228.x

[3] Reddy KV, Yedery RD, Aranha C (2004) Antimicrobial peptides: premises and promises. Int J Antimicrob Agents 24: 536–547. 15555874 10.1016/j.ijantimicag.2004.09.005

[4] Devine DA, Marsh PD, Percival RS, Rangarajan M, Curtis MA (1999) Modulation of antibacterial peptide activity by products of *Porphyromonas gingivalis* and *Prevotella* spp. Microbiology-Uk 145: 965–971. 10.1099/13500872-145-4-965 10220176

[5] Peschel A, Otto M, Jack RW, Kalbacher H, Jung G, et al (1999) Inactivation of the dlt operon in *Staphylococcus aureus* confers sensitivity to defensins, protegrins, and other antimicrobial peptides. J Biol Chem 274: 8405–8410. 10085071 10.1074/jbc.274.13.8405

[6] Shelton CL, Raffel FK, Beatty WL, Johnson SM, Mason KM (2011) Sap transporter mediated import and subsequent degradation of antimicrobial peptides in *Haemophilus* . PLoS Pathog 7: e1002360. 22072973 10.1371/journal.ppat.1002360 PMC3207918

[7] Nikaido H (1996) Multidrug efflux pumps of gram-negative bacteria. J Bacteriol 178: 5853–5859. 8830678 10.1128/jb.178.20.5853-5859.1996 PMC178438

[8] Joly S, Maze C, McCray PB Jr, Guthmiller JM (2004) Human beta-defensins 2 and 3 demonstrate strain-selective activity against oral microorganisms. J Clin Microbiol 42: 1024–1029. 15004048 10.1128/JCM.42.3.1024-1029.2004 PMC356847

[9] Murray BE (1990) The life and times of the *Enterococcus* . Clin Microbiol Rev 3: 46–65. 2404568 10.1128/cmr.3.1.46 PMC358140

[10] Al-Tahami K, Singh J (2007) Smart polymer based delivery systems for peptides and proteins. Recent Pat Drug Deliv Formul 1: 65–71. 19075875 10.2174/187221107779814113

[11] Shikanov A, Vaisman B, Krasko MY, Nyska A, Domb AJ (2004) Poly(sebacic acid-co-ricinoleic acid) biodegradable carrier for paclitaxel: In vitro release and in vivo toxicity. Journal of Biomedical Materials Research Part A 69A: 47–54. 10.1002/jbm.a.20101 14999750

[12] Shikanov A, Domb AJ (2006) Poly(sebacic acid-co-ricinoleic acid) biodegradable injectable in situ gelling polymer. Biomacromolecules 7: 288–296. 16398527 10.1021/bm050648+

[13] Shikanov A, Ezra A, Domb AJ (2005) Poly(sebacic acid-co-ricinoleic acid) biodegradable carrier for paclitaxel-effect of additives. Journal of Controlled Release 105: 52–67. 15955366 10.1016/j.jconrel.2005.02.018

[14] Slager J, Tyler B, Shikanov A, Domb AJ, Shogen K, et al (2009) Local Controlled Delivery of Anti-Neoplastic RNAse to the Brain. Pharmaceutical Research 26: 1838–1846. 19415468 10.1007/s11095-009-9893-3 PMC3085080

[15] Vaisman B, Motiei M, Nyska A, Domb AJ (2010) Biocompatibility and safety evaluation of a ricinoleic acid-based poly(ester-anhydride) copolymer after implantation in rats. Journal of Biomedical Materials Research Part A 92A: 419–431. 10.1002/jbm.a.32342 19191319

[16] Makovitzki A, Avrahami D, Shai Y (2006) Ultrashort antibacterial and antifungal lipopeptides. Proc Natl Acad Sci U S A 103: 15997–16002. 17038500 10.1073/pnas.0606129103 PMC1635116

[17] Papo N, Oren Z, Pag U, Sahl HG, Shai Y (2002) The consequence of sequence alteration of an amphipathic alpha-helical antimicrobial peptide and its diastereomers. J Biol Chem 277: 33913–33921. 12110678 10.1074/jbc.M204928200

[18] Krasko MY, Shikanov A, Ezra A, Domb AJ (2003) Poly(ester anhydride)s prepared by the insertion of ricinoleic acid into poly(sebacic acid). Journal of Polymer Science Part a-Polymer Chemistry 41: 1059–1069.

[19] Soren L, Nilsson M, Nilsson LE (1995) Quantitation of Antibiotic Effects on Bacteria by Bioluminescence, Viable Counting and Quantal Analysis. Journal of Antimicrobial Chemotherapy 35: 669–674. 7592180 10.1093/jac/35.5.669

[20] Beyth N, Yudovin-Farber I, Perez-Davidi M, Domb AJ, Weiss EI (2010) Polyethyleneimine nanoparticles incorporated into resin composite cause cell death and trigger biofilm stress in vivo. Proc Natl Acad Sci U S A 107: 22038–22043. 21131569 10.1073/pnas.1010341107 PMC3009770

[21] Stuart CH, Schwartz SA, Beeson TJ, Owatz CB (2006) *Enterococcus faecalis*: its role in root canal treatment failure and current concepts in retreatment. J Endod 32: 93–98. 16427453 10.1016/j.joen.2005.10.049

[22] Lee JK, Chang SW, Perinpanayagam H, Lim SM, Park YJ, et al (2013) Antibacterial Efficacy of a Human beta-Defensin-3 Peptide on Multispecies Biofilms. Journal of Endodontics 39: 1625–1629. 24238461 10.1016/j.joen.2013.07.035

[23] Lee JK, Park YJ, Kum KY, Han SH, Chang SW, et al (2013) Antimicrobial efficacy of a human -defensin-3 peptide using an *Enterococcus faecalis* dentine infection model. International Endodontic Journal 46: 406–412. 23078156 10.1111/iej.12002

[24] Lee SH, Baek DH (2012) Antibacterial and Neutralizing Effect of Human beta-Defensins on *Enterococcus faecalis* and *Enterococcus faecalis* Lipoteichoic Add. Journal of Endodontics 38: 351–356. 22341073 10.1016/j.joen.2011.12.026

[25] Swenson JM, Clark NC, Sahm DF, Ferraro ML, Doern G, et al (1995) Molecular Characterization and Multilaboratory Evaluation of *Enterococcus-Faecalis* Atcc-51299 for Quality-Control of Screening-Tests for Vancomycin and High-Level Aminoglycoside Resistance in *Enterococci* . J Clin Microbiol 33: 3019–3021. 8576364 10.1128/jcm.33.11.3019-3021.1995 PMC228625

[26] Jain JP, Sokolsky M, Kumar N, Domb AJ (2008) Fatty acid based biodegradable polymer. Polymer Reviews 48: 156–191.

[27] Krishna OD, Kiick KL (2010) Protein- and peptide-modified synthetic polymeric biomaterials. Biopolymers 94: 32–48. 20091878 10.1002/bip.21333 PMC4437713

